# Epidermis-Specific Metabolic Engineering of Sesquiterpene Formation in Tomato Affects the Performance of Potato Aphid *Macrosiphum euphorbiae*

**DOI:** 10.3389/fpls.2021.793313

**Published:** 2021-12-22

**Authors:** Fumin Wang, Yong-Lak Park, Michael Gutensohn

**Affiliations:** Division of Plant and Soil Sciences, Davis College of Agriculture, Natural Resources and Design, West Virginia University, Morgantown, WV, United States

**Keywords:** sesquiterpenes, metabolic engineering, multicistronic expression constructs, prenyl transferases, terpene synthases, epidermis, *Solanum lycopersicum*, potato aphid

## Abstract

Tomato produces a number of terpenes in their glandular trichomes that contribute to host plant resistance against pests. While glandular trichomes of cultivated tomato *Solanum lycopersicum* primarily accumulate a blend of monoterpenes, those of the wild tomato species *Solanum habrochaites* produce various sesquiterpenes. Recently, we have identified two groups of sesquiterpenes in *S. habrochaites* accessions that negatively affect the performance and choice behavior of the potato aphid (*Macrosiphum euphorbiae*). Aphids are piercing-sucking herbivores that use their mouthpart to penetrate and probe plant tissues in order to ultimately access vascular tissue and ingest phloem sap. Because secondary metabolites produced in glandular trichomes can affect the initial steps of the aphid feeding behavior, introducing the formation of defensive terpenes into additional plant tissues *via* metabolic engineering has the potential to reduce tissue penetration by aphids and in consequence virus transmission. Here, we have developed two multicistronic expression constructs based on the two sesquiterpene traits with activity toward *M. euphorbiae* previously identified in *S. habrochaites*. Both constructs are composed of sequences encoding a prenyl transferase and a respective *S. habrochaites* terpene synthase, as well as enhanced green fluorescent protein as a visible marker. All three coding sequences were linked by short nucleotide sequences encoding the foot-and-mouth disease virus 2A self-processing oligopeptide which allows their co-expression under the control of one promoter. Transient expression of both constructs under the epidermis-specific *Arabidopsis CER5*-promoter in tomato leaves demonstrated that formation of the two sets of defensive sesquiterpenes, β-caryophyllene/α-humulene and (−)-*endo*-α-bergamotene/(+)-α-santalene/(+)-*endo*-β-bergamotene, can be introduced into new tissues in tomato. The epidermis-specific transgene expression and terpene formation were verified by fluorescence microscopy and tissue fractionation with subsequent analysis of terpene profiles, respectively. In addition, the longevity and fecundity of *M. euphorbiae* feeding on these engineered tomato leaves were significantly reduced, demonstrating the efficacy of this novel aphid control strategy.

## Introduction

In nature, plants are an integral part of a complex system of antagonistic and mutualistic biotic interactions. Due to their sessile lifestyle, plants have adapted to the resulting challenges by evolving specific strategies for the defense against attacking as well as the attraction of beneficial organisms. Many of these strategies include the formation of secondary metabolites, such as volatile organic compounds (VOCs), that belong to three major categories: phenylpropanoids/benzenoids, fatty acid derivatives, and terpenes (Dudareva et al., 2013). Volatile mono- and sesquiterpenes synthesized by plants are known to contribute to the direct and indirect defense against phytophagous insects. While terpenes accumulated in plant tissues can be toxic to biting-chewing and piercing-sucking herbivores, their emission into the surrounding atmosphere can act repellent to these herbivores and attractive toward their natural enemies (Degenhardt et al., 2003; Gershenzon and Dudareva, 2007; Unsicker et al., 2009). To facilitate their role in the antagonistic interactions, terpenes are often produced in specific plant tissues including internal ducts, extracellular cavities, and glandular trichomes (Gershenzon and Dudareva, 2007; Zulak and Bohlmann, 2010). Volatile terpenes, like all other terpenoids, are synthesized from the building blocks isopentenyl diphosphate (IPP) and its isomer dimethylallyl diphosphate (DMAPP), which in plants originate from two parallel pathways: the mevalonic acid (MVA) pathway and the methylerythritol phosphate (MEP) pathway (Ashour et al., 2010; Hemmerlin et al., 2012). IPP and DMAPP are subsequently utilized by prenyl transferases to form larger *trans* and *cis* prenyl diphosphate intermediates. While geranyl diphosphate (GPP) and its *cis* isomer neryl diphosphate (NPP) are used for monoterpene synthesis, *trans*- and *cis*-farnesyl diphosphate (*E*,*E*- and *Z*,*Z*-FPP) serve as precursors for sesquiterpenes. The formation of terpenes is then catalyzed by terpene synthases (TPSs) which utilize one or several of the prenyl diphosphate substrates, and frequently have the ability to form multiple different terpene products from one prenyl diphosphate substrate (Degenhardt et al., 2009).

While defensive terpene traits are under positive selection pressure to ensure survival in wild plants, it appears that they have been compromised in cultivated crop plants since selective breeding has favored other agronomic traits (Köllner et al., 2008). Domestication and breeding have also resulted in the introduction of strong genetic bottlenecks in cultivated tomato (*Solanum lycopersicum*) which suffers from a higher susceptibility to various pests compared to wild tomato species (Bai and Lindhout, 2007). Although glandular trichomes are present on vegetative tissues of both cultivated and wild tomato species, these differ significantly in their profile of volatile terpenes. In *S. lycopersicum*, they primarily produce a blend of monoterpenes and only small amounts of a few sesquiterpenes (Schilmiller et al., 2009). In contrast, the systematic characterization of glandular trichome-derived terpenes in different accessions of the wild tomato species *Solanum habrochaites* (previously *Lycopersicon hirsutum*), for example, revealed several chemotypes characterized by the dominant formation of distinct blends of sesquiterpenes (Gonzales-Vigil et al., 2012), of which most are not present in *S. lycopersicum*. Numerous studies have shown that glandular trichome-derived terpenes found in *S. lycopersicum* as well as the *S. habrochaites* accessions have repellent and toxic activity against different biting-chewing herbivores including Colorado potato beetle (*Leptinotarsa decemlineata*), tobacco hornworm (*Manduca sexta*), tomato fruitworm (*Helicoverpa zea*), and beet armyworm (*Spodoptera exigua*; Carter et al., 1989a,b; Frelichowski and Juvik, 2001; Kang et al., 2010a,b; Tian et al., 2012; Gutensohn et al., 2014). In contrast, relatively little is known about the potential effects of glandular trichome-derived terpenes in cultivated and wild tomato on piercing-sucking herbivores, such as whiteflies, spider mites, and aphids. The sesquiterpene 7-*epi*-zingiberene and some of its derivatives produced in the glandular trichomes of several *S. habrochaites* accessions were shown to have repellent and/or toxic activity against silverleaf whiteflies (*Bemisia tabaci*) and two spotted-spider mites (*Tetranychus urticae*) (Bleeker et al., 2011a, 2012; Dawood and Snyder, 2020; Zabel et al., 2021). Utilizing a collection of *S. habrochaites* accessions with different glandular trichome-derived terpenes, we have recently identified two groups of sesquiterpenes that affect the potato aphid (*Macrosiphum euphorbiae*) (Wang et al., 2020). *S. habrochaites* accessions producing β-caryophyllene and α-humulene, or (−)-*endo*-α-bergamotene, (+)-α-santalene, and (+)-*endo*-β-bergamotene, respectively, not only reduced the aphid longevity and fecundity significantly, but also had repellent activity against *M. euphorbiae*. Remarkably, by utilizing two tomato trichome mutants, *hairless* and *odorless-2*, that are differently affected in mono- and sesquiterpene production (Kang et al., 2010a,b), we demonstrated that the relatively small amounts of β-caryophyllene and α-humulene in glandular trichomes of cultivated tomato still have some effect on the performance of *M. euphorbiae* (Wang et al., 2021). However, the same analysis also suggested a role of the highly abundant TPS20-derived monoterpenes in the attraction of aphids to cultivated tomato, which was further confirmed using a mixture of pure monoterpenes (Wang et al., 2021).

*Macrosiphum euphorbiae* is an important agricultural pest that causes economic losses to horticultural crop production including tomato (Tomescu and Negru, 2003). Damage is caused not only by the direct feeding of *M. euphorbiae*, but even more by the transmission of multiple non-persistent and persistent viruses for which these aphids serve as vectors (Blackman and Eastop, 2000; van Emden and Harrington, 2007). This results in reduced crop yield and quality, and often plant death even at low levels of aphid infestation (Lange and Bronson, 1981). Current aphid control strategies utilizing synthetic insecticides are increasingly inefficient due to emerging resistances and avoidance behavior (Silva et al., 2012; Bass et al., 2014; Fray et al., 2014). Thus, introducing the production of the two groups of sesquiterpenes that we have identified in the *S. habrochaites* accessions and that affected choice behavior and performance of *M. euphorbiae* (Wang et al., 2020) into cultivated tomato represents a promising approach toward developing a sustainable aphid control strategy. One possible way for the introduction of these defensive terpene traits is the classical genetic approach by crossing *S. lycopersicum* with a respective *S. habrochaites* accession, followed by several backcrosses into the cultivated tomato background to create an introgression line carrying the terpene trait from *S. habrochaites*. Indeed, we have used a near isogenic line containing a small *S. habrochaites* introgression on chromosome 8 (van der Hoeven et al., 2000) that carries the genes encoding the respective prenyl transferase and terpene synthase, and was found to produce (−)-*endo*-α-bergamotene, (+)-α-santalene and (+)-*endo*-β-bergamotene (Sallaud et al., 2009). Our assays performed with this line confirmed that introgression of (−)-*endo*-α-bergamotene/(+)-α-santalene/(+)-*endo*-β-bergamotene formation into the cultivated tomato background had successfully transferred this defensive trait affecting performance and choice behavior of *M. euphorbiae* (Wang et al., 2020). However, compared to the parental *S. habrochaites* accession the amounts of (−)-*endo*-α-bergamotene, (+)-α-santalene, and (+)-*endo*-β-bergamotene, and in consequence, the effects on *M. euphorbiae* were significantly lower in the near isogenic line (Wang et al., 2020) highlighting a potential limitation of this classical genetic approach. The observed difference in sesquiterpene production is likely due to a higher expression level of MVA and MEP pathway genes (Besser et al., 2009) and a larger storage capacity in the internal cavity of the glandular trichomes (Therezan et al., 2021) in the parental *S. habrochaites* accession compared to the introgression line. The described classical genetic approach limits the introduction of defensive terpene traits into cultivated tomato to the tissue(s) where the respective biosynthetic genes are expressed in wild tomato, for example, glandular trichomes. In contrast, metabolic engineering represents an efficient approach to introduce a respective biosynthetic pathway into a plant tissue and/or species naturally devoid of a terpene compound of interest. Multiple examples for successful metabolic engineering of terpenes in different plant species have been reported (summarized in Lange and Ahkami, 2013; Vickers et al., 2014) with three types of genes being used individually or in combination: MVA/MEP pathway, prenyl transferase, and TPS genes. Two of our previous metabolic engineering studies in tomato (Gutensohn et al., 2013, 2014) demonstrated that co-expression of prenyl transferases and respective TPSs utilizing the produced prenyl diphosphates resulted in the formation of significant amounts of the expected terpenes. To avoid potential negative effects on plant growth and performance recent engineering strategies utilized specific promoters to restrict the expression of terpene biosynthetic genes to particular plant tissues or organs (Lewinsohn et al., 2001; Davidovich-Rikanati et al., 2007, 2008; Morris et al., 2011; Bleeker et al., 2012; Borghi and Xie, 2016).

Aphids as piercing-sucking herbivores use their stylet, a specialized mouthpart, to ultimately access vascular tissue and ingest phloem sap. However, to achieve sustained phloem sap ingestion, the feeding behavior of aphids progresses through several stages (Powell et al., 2006): (i) pre-alighting behavior, (ii) initial plant contact and assessment of surface cues before stylet insertion, (iii) probing of the plant epidermis, (iv) stylet pathway activity, (v) sieve element puncture and salivation, and (vi) phloem acceptance and ingestion. Blocking or hindering these feeding behaviors could lead to the development of a novel aphid control strategy. Terpenes produced in glandular trichomes of tomato are likely only affecting the initial steps of the aphid feeding behavior. However, introducing the formation of defensive terpenes into additional plant tissues *via* metabolic engineering could have the potential to reduce or even eliminate plant tissue penetration by aphids and in consequence virus transmission. In this study, we have developed two multicistronic expression constructs based on the two sesquiterpene traits previously identified in *S. habrochaites* (Wang et al., 2020) that are composed of the coding sequences for prenyl transferases and respective *S. habrochaites* terpene synthases, as well as enhanced green fluorescent protein as a visible marker. Transient expression of both constructs under the epidermis-specific *CER5*-promoter in tomato leaves demonstrated that formation of the two sets of defensive sesquiterpenes, β-caryophyllene/α-humulene and (−)-*endo*-α-bergamotene/(+)-α-santalene/(+)-*endo*-β-bergamotene, can be introduced into new tissues in tomato. The epidermis-specific transgene expression and terpene formation were verified by fluorescence microscopy and tissue fractionation with subsequent analysis of terpene profiles, respectively. In addition, the longevity and fecundity of *M. euphorbiae* that fed on these engineered tomato leaves were significantly reduced, demonstrating the efficacy of this novel aphid control strategy.

## Materials and Methods

### Plant Material

Seeds of the tomato (*S. lycopersicum*) trichome mutant *odorless-2* (Kang et al., 2010a) were kindly provided by Dr. Gregg Howe (Michigan State University, MI, United States). Tomato plants used for *Agrobacterium* leaf infiltration and all subsequent analyses were grown from seeds in Sungro^®^ soil mixture (Sun Gro Horticulture, Agawam, MA, United States) in multi-trays (288 cells, 5 ml per cell), and seedlings were transplanted into 4-inch square pots. Plants were grown under a 16-h photoperiod in a climate-controlled growth room (23–25°C, 50–60% relative humidity) without pesticide application.

### Cloning and Plasmid Construction

For the cloning of the two multicistronic gene constructs under the control of a tissue-specific promoter, the binary vector pMCS:GW was used (obtained from the Arabidopsis Biological Resource Center, stock # CD3-1933) that contains a multiple cloning site upstream of the Gateway cassette (Michniewicz et al., 2015). To obtain the promoter region of the *AtCER5* gene (At1g51500; Pighin et al., 2004), a 2,614 base pair fragment was amplified by PCR from *Arabidopsis thaliana* genomic DNA using Taq polymerase (GenScript, Piscataway, NJ, United States) and a pair of oligonucleotides each carrying a specific restriction site (*AtCER5*-fwd-*Eco*RI: 5'-CGGAATTCTTTAGTTTGCTTG AGTTCTCATG-3'; *AtCER5*-rev-*Sal*I: 5'-GCGTCGACTGTTTT TGTTTGATCTTGAAAAAGATC-3'). The resulting PCR product was cloned into the vector pMD20 (Takara Bio, United States) and sequenced to verify its correct amplification. The *AtCER5* promoter fragment was subsequently excised from the pMD20 vector by *Eco*RI and *Sal*I digestion, and then ligated between the *Eco*RI and *Xho*I sites of the pMCS:GW vector.

The two multicistronic gene constructs were obtained by gene synthesis (Twist Bioscience, San Francisco, CA, United States). These gene constructs were flanked by attL-sequences and inserted into the Gateway Entry vector pTwist ENTR (Twist Bioscience, San Francisco, CA, United States). Both of these multicistronic constructs contained three consecutive coding regions encoding a prenyltransferase, a terpene synthase, and enhanced green fluorescent protein, respectively. Stop codons of the prenyl transferase and terpene synthase coding sequences were removed and instead 60-bp wild type F2A sequences (5'- CAGCTGTTGAATTTTGACCTT CTTAAGCTTGCGGGAGACGTCGAGTCCAACCCTGGG CCC-3'; Ryan et al., 1991; Kenneth et al., 2012) inserted such that the three coding regions were linked into one ORF. In addition, the 5'-UTR sequence of the *A. thaliana Farnesyl Diposphate Synthase 2 AtFPPS2* gene (At5g47770) was fused upstream of the start codon of the prenyl transferase coding sequence in both gene constructs.

Each of the two multicistronic gene constructs was transferred from the pTwist ENTR vector into the pMCS:GW vector carrying the *AtCER5* promoter fragment by performing a standard LR recombination reaction. One μl of the destination vector (150 ng/μl) and 0.5 μl of the entry vector (30 ng/μl) were mixed with 6.5 μl TE buffer and 2 μl LR Clonase II Plus enzyme mix (Invitrogen, Thermo Fisher Scientific), and incubated at 25°C for 1 h. After the addition of 1 μl of proteinase K (Invitrogen, Thermo Fisher Scientific), reactions were incubated at 37°C for 10 min. Aliquots of each LR reaction were transformed into competent *Escherichia coli* DH5α cells (Thermo Fisher Scientific) and colonies with recombinant plasmids selected on LB agar plates with kanamycin (100 μg/ml).

### Agrobacterium Leaf Infiltration

The resulting binary pMCS:GW vectors carrying the multicistronic gene constructs under the control of the *AtCER5* promoter were introduced into *Agrobacterium tumefaciens* (strain GV3101), and those were subsequently used for transient transformation *via* infiltration of tomato leaves as described previously (Norkunas et al., 2018). Selected *Agrobacterium* clones were grown in LB medium containing kanamycin (100 μg/ml), rifampicin (25 μg/ml), and gentamycin (50 μg/ml) to an OD_600_ of 0.8–1.0. Bacterial cultures were harvested by centrifugation and resuspended in infiltration buffer [10 mm MES-KOH (pH 5.7), 10 mm MgCl_2_ and 200 μm acetosyringone]. *Agrobacterium* suspensions were adjusted to an OD_600_ of 0.6–0.8 and further incubated at room temperature for 2 h prior to leaf infiltration. *Agrobacterium*-mediated transient transformation was subsequently conducted on the second and older true leaves of 4-week-old *odorless-2* tomato plants. The *Agrobacterium* suspension was injected into leaves from the abaxial side using a 5 ml plastic syringe (without hypodermic needle) thus infiltrating the intercellular spaces of the entire leaves. After the infiltration plants were covered by a humidity dome and continued to grow under a 16-h photoperiod at 23–25°C until further analysis.

### RNA Extraction and RT-PCR Analysis

Six days after the *Agrobacterium* infiltration total RNA was isolated from infiltrated tomato leaves as previously described (Eggermont et al., 1996). For the RT-PCR analyses, total RNA was pretreated with RNase-free DNase (New England Biolabs, Ipswich, MA, United States) and cDNA was synthesized using reverse transcriptase (Superscript II, Invitrogen, Carlsbad, CA, United States). To evaluate the expression of both multicistronic gene constructs, the cDNA was subsequently used for PCR utilizing three primer pairs specific for: *AtFPPS2* (*AtFPPS*-fwd: 5'-CGGATCTGAAATCAACCTTCCTCGAC-3'; *AtFPPS*-rev: 5'- CAATGCCTTAACTACCAACCAGGAGC-3'), *ShzFPPS* (*ShzFPPS*-fwd: 5' -CAAATTCACCTCTGACAGTGTCTGC-3'; *ShzFPPS*-rev: 5'-GTGTGTCCACCAAAACGTCTATGCC-3'), and *eGFP* (*eGFP*-fwd: 5'- CGACGTAAACGGCCACAAGTT CA-3'; *eGFP*-rev: 5'- ACTTGTACAGCTCGTCCATGCC-3'). The PCR conditions were as follows: 94°C for 5 min for one cycle, followed by 35 cycles of 95°C for 60 s, 57°C for 60 s and 72°C for 60 s, and a final extension at 72°C for 10 min. The amplification products were separated by agarose gel electrophoresis, stained with GelRed^®^ (Biotium, United States), and analyzed using the ChemiDoc Gel Imaging System and Image Lab 5.1 software (Bio-Rad, Hercules, CA, United States).

### Fluorescence Microscopy

Four to five days after *Agrobacterium* infiltration tomato leaves were collected, and cross- and surface sections were prepared. Leaf cross- and surface sections were analyzed with a Zeiss Axio Imager M1 compound microscope (Zeiss, Oberkochen, Germany) equipped with an X-Cite series 120Q fluorescent illuminator. Fluorescence microscopy was performed using a 485/20 excitation filter in combination with a green filter cube, and a 545/25 excitation filter in combination with a red filter cube for the analysis of GFP fluorescence and chlorophyll autofluorescence, respectively. All images were captured using a monochrome AxioCam MRm camera mounted on the Imager M1 microscope and further processed with the AxioVision software. Composite images from GFP and chlorophyll fluorescence microscopy were overlaid using the ImageJ software (National Institutes of Health).^1^

### Extraction of Terpenes From Whole Tomato Leaves

Tomato leaves were collected at different time points (0, 3, 6, 9, 12, and 15 days) after the *Agrobacterium* infiltration. Leaves were photographed, and their surface areas were determined using the ImageJ software (National Institutes of Health, see footnote 1). Leaves were stirred in 50 ml of methyl *tert*-butyl ether (MTBE) for 20 min to extract terpenes as described previously (Gutensohn et al., 2013). After removing the leaves, extracts were concentrated under a gentle stream of nitrogen gas to a volume of 200 μl and centrifuged for further purification. For the analysis of terpene profiles, extracts were transferred into GC vials and supplemented with 3.33 μg of naphthalene as an internal standard.

### Extraction of Terpenes From Isolated Trichomes, Epidermis, Vasculature, and Mesophyll From Tomato Leaves

To further investigate the accumulation of terpenes in different leaf tissues, glandular trichomes, epidermis, vasculature, and mesophyll were isolated from tomato leaves 15 days after *Agrobacterium* infiltration. Type VI glandular trichomes were collected from tomato leaves as described previously (Schilmiller et al., 2009) using a stretched Pasteur pipette under a stereomicroscope (SZ-ST, Olympus, Tokyo, Japan). A total of 200 trichomes from each leaf sample were accumulated into 10 ml of MTBE for the extraction of terpenes. For the isolation of the other leaf tissues, a protocol was adapted from previous studies (Endo et al., 2016; Svozil et al., 2016). Entire tomato leaflets were sandwiched between two layers of clear scotch tape, and the abaxial leaf epidermis was peeled by gently pulling off one tape. The tape with the attached abaxial leaf epidermis was immediately transferred into 10 ml MTBE for terpene extraction. The other layer of tape with the remainder of the leaf was transferred into a 50 ml tube containing 15 ml of enzyme solution [1.00% (w/v) cellulase R-10, 0.25% (w/v) macerozyme R-10, 0.4 M mannitol, 8 mm CaCl_2_, and 5 mm MES-KOH, pH 5.7]. After a 10 min incubation, the leaf vasculature was isolated by using a dissecting needle and transferred into 10 ml MTBE. The remaining leaf tissue was further digested for an additional 15 min for removing mesophyll cells into the enzyme solution. Subsequently, the tape with the attached adaxial leaf epidermis was transferred into the vial which already contained the tape with the abaxial leaf epidermis. The mesophyll cells isolated by the enzyme treatment were further purified using a 30 μm cell strainer (pluriSelect, Leipzig, Germany), centrifuged at 1,000 *g* for 5 min, and resuspended into 10 ml MTBE. All isolated leaf tissue fractions were allowed to shake in MTBE at 100 rpm for 30 min for terpene extraction. All extracts were concentrated under a gentle stream of nitrogen gas to a volume of 200 μl and centrifuged for further purification. Concentrated and purified extracts were transferred into GC vials and supplemented with 3.33 μg of naphthalene as an internal standard.

### Gas Chromatography–Mass Spectrometry Analysis

All extracts from entire tomato leaves and leaf tissue fractions were analyzed by combined gas chromatography–mass spectrometry (GC–MS) using a TRACE 1310 gas chromatograph system linked to a TSQ 8000 Triple Quadrupole mass spectrometer (Thermo Fisher Scientific, Waltham, MA, United States). Two μl of each sample was injected under a spitless mode, volatilized at 220°C, and then separated on a TraceGOLD TG-5MS GC column (30 m length, 0.25 mm I.D., and 0.25 μm film; Thermo Fisher Scientific, Pittsburgh, PA, United States). The initial column temperature was held at 40°C for 3 min and then ramped at 5°C/min to 120°C, 10°C/min to 180°C, and 20°C/min to 300°C which was maintained for 2 min. The helium carrier gas flow was 1.3 ml/min. All samples were analyzed using the total ion chromatogram (TIC) mode. Individual terpene compounds were identified by comparing their mass spectra (15–300 m/z) with those deposited in the NIST/EPA/NIH Mass Spectral Library (NIST11; National Institute of Standards and Technology NIST, Scientific Instrument Services, Inc., NJ, United States),^2^ as well as those reported previously (Sallaud et al., 2009). Terpenes identified in the leaf and leaf tissue extracts were quantified using a previously determined average response factor for sesquiterpenes (Wang et al., 2020) in combination with the internal naphthalene standard.

### Aphid Culture

A potato aphid (*M. euphorbiae*) colony was established from apterae collected in the WVU Evansdale Greenhouse (Morgantown, WV, United States). To avoid experience on tomato plants prior to the non-choice assays on *odorless-2* plants, aphids were allowed to reproduce parthenogenetically on potted potato plants in an insect rearing room under a 16-h photoperiod at 20–22°C. The aphid species was confirmed through barcode sequencing.

### Aphid Non-Choice Assays

The performance (longevity and fecundity) of *M. euphorbiae* apterae on agroinfiltrated leaves of *odorless-2* tomato plants was determined in a climate-controlled growth room (23–25°C, 50–60% relative humidity, and 16-h photoperiod) as described previously (Eichele-Nelson et al., 2018; Wang et al., 2020). Before the assay apterae (*F*_0_) reared on potato plants were introduced on the leaf surface of *odorless-2* plants. Four days after the introduction three neonate *F*_1_ nymphs (considered as 1 day old) were carefully transferred to the surface of a young *odorless-2* leaf (second or third fully expanded leaf) which had been infiltrated with *Agrobacterium* 2 days earlier, and subsequently enclosed in a clip cage (BioQuip Products, Rancho Dominguez, CA, United States) that was attached to the leaf. Four tomato plants each with three clip cages attached (in total 12 clip cages) were used for each of the treatments (two expression constructs and one control). Over the course of the experiment *F*_2_ nymphs and exuviae were removed daily from the clip cages. The longevity of *F*_1_ nymphs and their fecundity represented by the number of *F*_2_ nymphs in each cage were recorded. The longevity and fecundity of aphids were analyzed by one-way ANOVA followed by multiple comparisons using Tukey’s HSD (*α* = 0.05).

## Results

### Design of Two Multicistronic Expression Constructs for Epidermis-Specific Engineering of Sesquiterpene Formation

**Figure 1:**
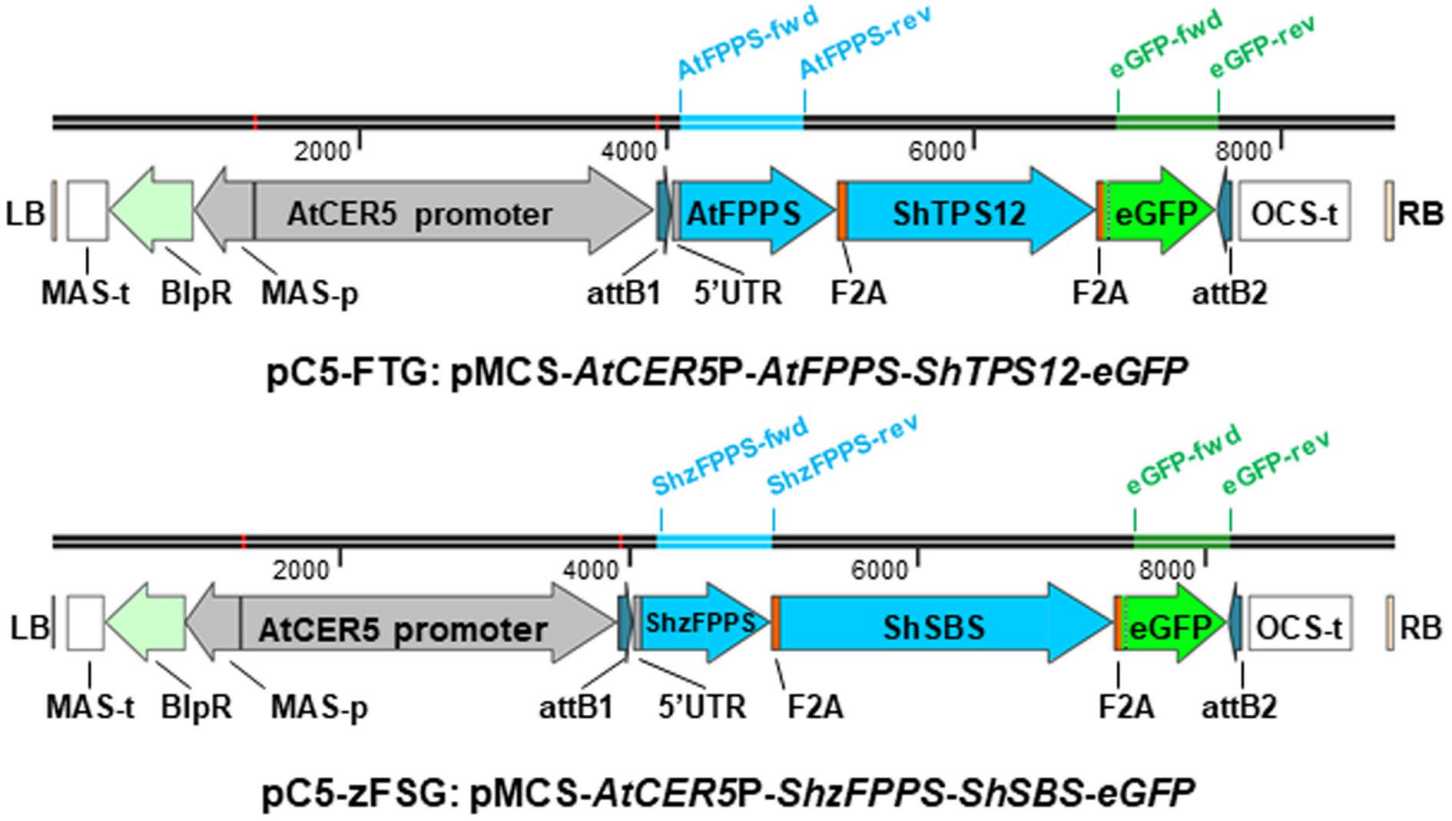
Schematic representation of multicistronic expression constructs for epidermis-specific engineering of sesquiterpene formation. Two expression constructs were designed within the T-DNA, indicated by the left (LB) and right (RB) borders, of the binary pMCS vector. Both synthetic expression constructs are put under the control of an *AtCER5* promoter sequence and inserted between the gateway attachments sites (attB1 and attB2). Each of the multicistronic expression constructs contains the coding sequences for three proteins: a prenyl transferase (AtFPPS or ShzFPPS), a terpene synthase (ShTPS12 or ShSBS), and enhanced green fluorescent protein (eGFP). The three individual coding sequences within both multicistronic expression constructs are linked by a short nucleotide sequence encoding the self-processing foot-and-mouth disease virus 2A oligopeptide (F2A). Other elements located within the T-DNA are the octopine synthase terminator (OCS-t), mannopine synthase promoter (MAS-p), and terminator (MAS-t) and phosphinothricin acetyltransferase (BlpR). The bars above the pC5-FTG and pC5-zFSG constructs indicate their size (in base pairs) and the location of the three primer pairs used for RT-PCR analysis of *AtFPPS*, *ShzFPPS*, and *eGFP* expression.

To engineer formation of the two groups of sesquiterpenes with activity against *M. euphorbiae* that we have identified in *S. habrochaites* (Wang et al., 2020) into the epidermis of *S. lycopersicum*, we have designed two multicistronic expression constructs (Figure 1) in the binary vector pMCS:GW (Michniewicz et al., 2015). Both constructs were put under the control of the promoter of the *AtCER5* gene (At1g51500; Figure 1), encoding a plasma membrane localized ABC transporter involved in the export of cuticular wax, that was shown to direct epidermis-specific expression (Pighin et al., 2004). To achieve high levels of terpene formation, we opted to co-express each of the selected *S. habrochaites* terpene synthases together with a respective prenyl transferase which assures sufficient availability of the required prenyl diphosphate substrate in the correct subcellular compartment. Despite the significant difference in β-caryophyllene/α-humulene formation between cultivated and wild tomato accessions (Wang et al., 2020), in both tomato species these sesquiterpenes are produced by Terpene Synthase 12 (TPS12) utilizing *E*,*E*-FPP as substrate (Schilmiller et al., 2010; Bleeker et al., 2011b). Thus, for the design of the respective expression construct (Figure 1), the coding sequence for the *S. habrochaites Terpene Synthase 12* (*ShTPS12*, GenBank accession JN402389; Bleeker et al., 2011b) was paired with that for the *A. thaliana Farnesyl Diphosphate Synthase 2* (*AtFPPS2*, At4g17190; Keim et al., 2012). The terpene synthase responsible for the formation of (−)-*endo*-α-bergamotene/(+)-α-santalene/(+)-*endo*-β-bergamotene in some *S. habrochaites* accessions, Santalene and Bergamotene Synthase (ShSBS), accepts *Z*,*Z*-FPP as substrate which is synthesized by *cis*-Farnesyl Diphosphate Synthase (ShzFPPS; Sallaud et al., 2009). In contrast to AtFPPS2 and ShTPS12, which are localized in the cytosol, ShzFPPS and ShSBS both carry N-terminal transit peptides that target them toward plastids. Thus, for the design of the second expression construct (Figure 1), the coding sequences of *ShSBS* (GenBank accession FJ194970) and *ShzFPPS* (GenBank accession FJ194969) (Sallaud et al., 2009) were paired. To obtain coordinated and stable expression of the multiple transgenes under the control of the *AtCER5* promoter, the open reading frames encoding the terpene synthase and prenyl transferase in both expression constructs (Figure 1) were linked by a short 60 bp nucleotide sequence encoding the foot-and-mouth disease virus 2A oligopeptide (F2A; Ryan et al., 1991; Kenneth et al., 2012). This F2A sequence represents a self-processing peptide that *via* a ribosome skipping mechanism during the translation process leads to the separation between the upstream polypeptide ending with the C-terminal 2A sequence and the next translation product downstream (Ryan and Flint, 1997; Donnelly et al., 2001). As third part, the coding region of *enhanced green fluorescent protein* (*eGFP*) was added to both multicistronic expression constructs (Figure 1) and was likewise linked by an F2A sequence to the 3' end of the terpene synthases, *ShTPS12* and *ShSBS*, respectively. In summary, both constructs (Figure 1), the pC5-FTG construct (*AtCER5*P-*AtFPPS*-*ShTPS12*-*eGFP*) and the pC5-zFSG construct (*AtCER5*P-*ShzFPPS*-*ShSBS*-*eGFP*), will result in the formation of three separate proteins upon expression *in planta*: a prenyl transferase and terpene synthase pair catalyzing the synthesis of the desired sesquiterpenes, as well as eGFP that will serve as a visual marker of the epidermis-specific expression.

### Transient Expression of the Multicistronic Constructs in Tomato Leaves

**Figure 2:**
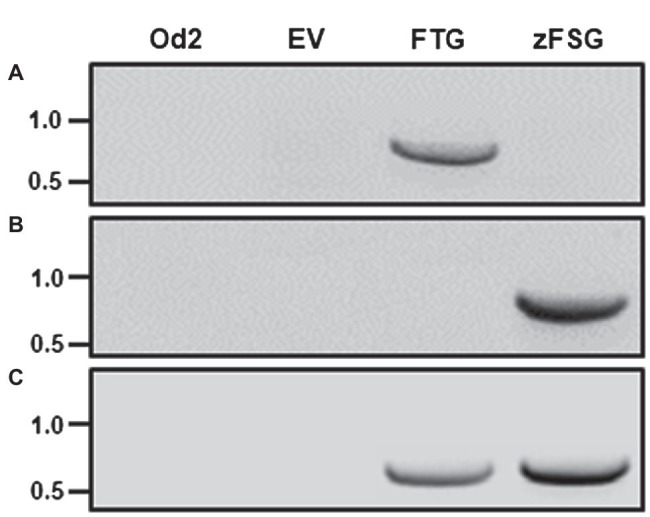
Transient expression of the multicistronic constructs in tomato leaves. Leaves of the tomato *odorless-2* mutant were infiltrated with *Agrobacterium* carrying the pC5-FTG construct, the pC5-zFSG construct, or the empty pMCS vector (EV). Transcript levels in *Agrobacterium* infiltrated leaves and *odorless-2* control (Od2) leaves were analyzed by RT-PCR utilizing *AtFPPS* **(A)**, *ShzFPPS* **(B)**, and *eGFP* **(C)** specific primer pairs (see Figure 1 for location). The amplification products indicating *AtFPPS* (788 bp), *ShzFPPS* (794 bp), and *eGFP* (656 bp) expression were separated by agarose gel electrophoresis (size marker in kb indicated with each panel).

We tested the function of the newly designed multicistronic expression constructs by infiltrating leaves of cultivated tomato with *Agrobacterium* carrying the pMCS binary vector with the inserted pC5-FTG and pC5-zFSG constructs (Figure 1), respectively, as well as the empty pMCS vector as a negative control. For these transient transformation assays, we used the tomato trichome mutant *odorless-2* (Kang et al., 2010a) since it is deficient in the formation of TPS20-derived monoterpenes and TPS12-derived sesquiterpenes that are naturally found in *S. lycopersicum* trichomes and thus could interfere with the analysis of the engineered sesquiterpenes. To determine the expression of both multicistronic constructs upon transient transformation of the tomato leaves reverse transcription-PCR (RT-PCR) analyses were performed using primer pairs (Figure 1) specific for the first coding region (*AtFPPS* and *ShzFPPS*) and the third coding region (*eGFP*) in the pC5-FTG and pC5-zFSG constructs, respectively. An *AtFPPS* specific 788 base pair cDNA fragment could be amplified from leaves infiltrated with *Agrobacterium* carrying the pC5-FTG construct (Figure 2A). However, this *AtFPPS* fragment was not detected with untransformed *odorless-2* control leaves or leaves infiltrated with *Agrobacterium* carrying the empty pMCS vector and the pC5-zFSG construct, respectively. In contrast, a *ShzFPPS*-specific 794 base pair cDNA fragment was only amplified from leaves infiltrated with *Agrobacterium* carrying the pC5-zFSG construct (Figure 2B) but was absent from the *odorless-2* and empty vector controls as well as leaves infiltrated with *Agrobacterium* carrying the pC5-FTG construct. The *eGFP*-specific 656 base pair cDNA fragment could be amplified from leaves infiltrated with *Agrobacterium* carrying either the pC5-FTG or pC5-zFSG construct (Figure 2C) but was not found with the *odorless-2* and empty vector controls. The fact that transcripts of the prenyl transferases and *eGFP* representing the first and last coding region in both constructs were detected by RT-PCR upon the transient transformation of tomato leaves (Figure 2) suggests that the entire multicistronic constructs were expressed under the control of the *AtCER5* promoter.

### Epidermis-Specific Expression of the Multicistronic Constructs

**Figure 3:**
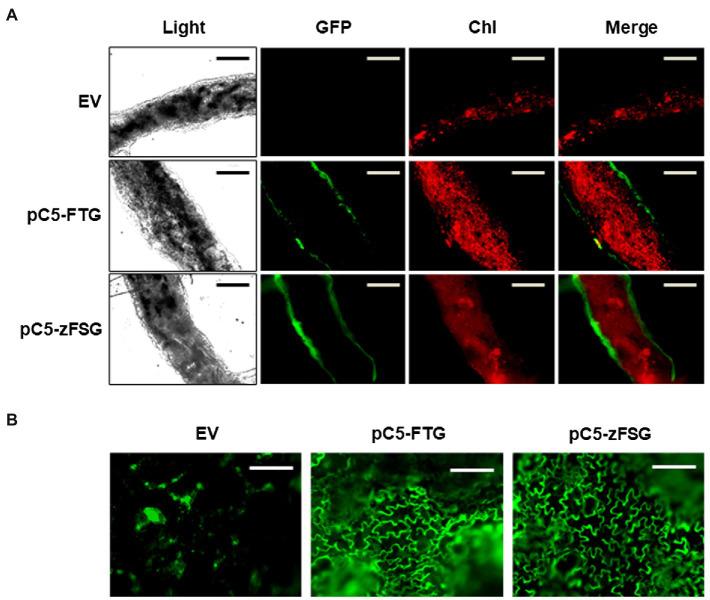
Tissue-specific expression of the multicistronic constructs. Cross-sections **(A)** and surface sections **(B)** of tomato leaves infiltrated with *Agrobacterium* carrying the pC5-FTG construct, the pC5-zFSG construct, or the empty pMCS vector (EV) were analyzed by light and confocal laser scanning microscopy. Panels show fluorescence of green fluorescent protein (GFP) and chlorophyll autofluorescence (Chl). Scale bars represent 100 μm.

While the RT-PCR analysis (Figure 2) in general demonstrated expression of the multicistronic constructs in tomato leaves upon transient transformation, the tissue specificity of their expression under the control of the *AtCER5* promoter remained to be shown. Toward this goal, we performed confocal fluorescence microscopy of tomato leaves that had been infiltrated with *Agrobacterium* carrying the empty pMCS vector, pC5-FTG construct, and pC5-zFSG construct, respectively, to determine the tissue-specific accumulation of eGFP which is encoded in both multicistronic constructs. When cross-sections of tomato leaves were analyzed, GFP fluorescence was exclusively detected in both epidermal layers of leaves transiently transformed with the pC5-FTG and pC5-zFSG constructs (Figure 3A), while no respective fluorescence was observed with the empty vector control. Moreover, the GFP fluorescence did not overlap with the chlorophyll fluorescence detected in the chloroplast containing parenchyma cells of the leaf cross-sections (Figure 3A). In addition, we analyzed surface sections of the transiently transformed tomato leaves by fluorescence microscopy to further verify the expression of *eGFP* in epidermis cells. Upon the transient expression of the pC5-FTG and pC5-zFSG constructs in tomato leaves, GFP fluorescence could be observed in the cytosolic rim of the epidermal pavement cells (Figure 3B). The results of these fluorescence microscopy analyses not only provide further evidence that the entire pC5-FTG and pC5-zFSG constructs including *eGFP* are expressed, but also indicate that their expression under the *AtCER5* promoter is indeed restricted to the epidermis of transformed tomato leaves.

### Sesquiterpene Formation in the Epidermis of Tomato Leaves Transiently Expressing the pC5-FTG and pC5-zFSG Constructs

**Figure 4:**
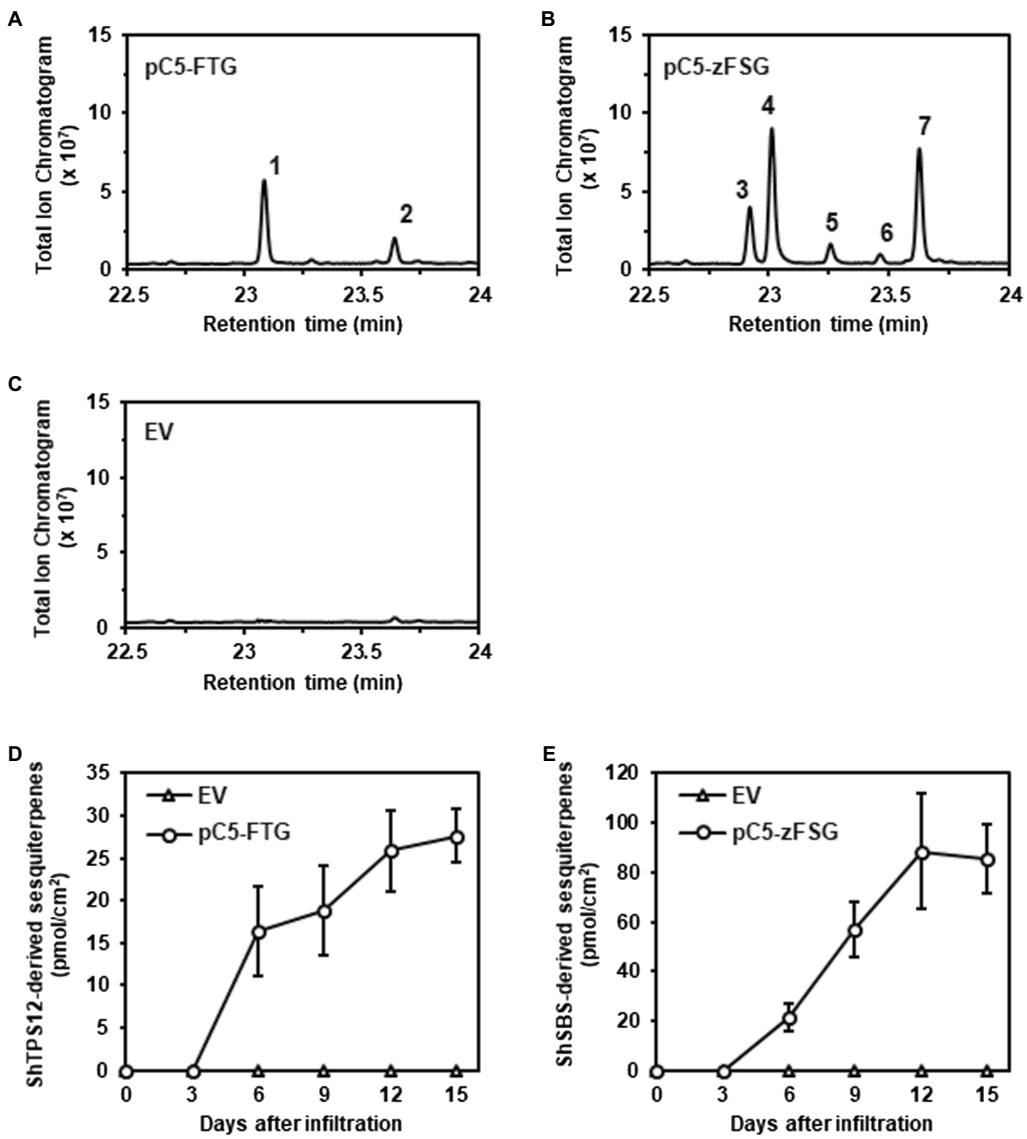
Accumulation of ShTPS12- and ShSBS-derived sesquiterpenes in tomato leaves expressing the multicistronic constructs. Terpenes were extracted from tomato leaves infiltrated with *Agrobacterium* carrying the pC5-FTG construct **(A)**, the pC5-zFSG construct **(B)**, or the empty pMCS vector (EV) **(C)** and were analyzed by GC–MS (total ion chromatograms are shown). ShTPS12-derived sesquiterpenes: 1, β-caryophyllene; 2, α-humulene. ShSBS-derived sesquiterpenes: 3, (−)-*endo*-α-bergamotene; 4, (+)-α-santalene; 5, (−)-*exo*-α-bergamotene; 6, (−)-*epi*-β-santalene; and 7, (+)-*endo*-β-bergamotene. The total amounts (pmol/cm^2^ leaf area) of ShTPS12-derived **(D)** and ShSBS-derived **(E)** sesquiterpenes were determined in tomato leaves at different time points after the *Agrobacterium* infiltration. Data are means ± SEM (*n* = 3).

To determine if the transient expression of the multicistronic constructs, both encoding pairs of prenyl transferases and terpene synthases, in the leaf epidermis, resulted in the formation of the expected sesquiterpenes, tomato leaves were extracted with methyl *tert*-butyl ether (MTBE) 15 days after *Agrobacterium* infiltration. The subsequent analysis of the leaf extracts by combined gas chromatography–mass spectrometry (GC–MS) demonstrated that leaves of the *odorless-2* tomato mutant expressing the pC5-FTG construct had accumulated the expected ShTPS12 products β-caryophyllene and α-humulene (Figure 4A; Supplementary Figure 1). A similar analysis of leaves infiltrated with *Agrobacterium* carrying the empty pMCS vector found no β-caryophyllene and α-humulene accumulation (Figure 4C) which is in line with the previous characterization of the *odorless-2* tomato mutant (Kang et al., 2010a; Wang et al., 2021) showing the absence of these two sesquiterpenes in this trichome mutant. In contrast, the analysis of tomato leaves expressing the pC5-zFSG construct revealed a different profile of accumulated terpenes (Figure 4B; Supplementary Figure 2) including (−)-*endo*-α-bergamotene, (+)-α-santalene, (−)-*exo*-α-bergamotene, (−)-*epi*-β-santalene, and (+)-*endo*-β-bergamotene. These five sesquiterpenes have been observed previously in *in vitro* enzyme assays as well as in a transgenic tobacco line as products of ShSBS when *Z*,*Z*-FPP was provided as substrate by ShzFPPS (Sallaud et al., 2009).

The quantitative analysis of the terpene accumulation in tomato leaves expressing the pC5-FTG and pC5-zFSG constructs showed that the ShTPS12- and ShSBS-derived sesquiterpene products, respectively, could be detected for the first time 6 days after the *Agrobacterium* infiltration (Figures 4D,E; Supplementary Table 1). Subsequently, the amounts of the sesquiterpene products in the tomato leaves continued to increase until 12 days after the *Agrobacterium* infiltration and appeared to remain constant afterward (Figures 4D,E; Supplementary Table 1). Remarkably, the total amount of sesquiterpenes produced after 15 days in leaves expressing the plastid localized ShzFPPS and ShSBS were 3.1-fold higher than in leaves expressing the cytosolic AtFPPS and ShTPS12 (Figures 4D,E; Supplementary Table 1).

To further verify the tissue specificity of the novel metabolic engineering approach described here, we studied the accumulation of sesquiterpenes in different tissues of tomato leaves transiently expressing the pC5-FTG and pC5-zFSG constructs. Fifteen days after *Agrobacterium* infiltration tomato leaves were separated into epidermis, mesophyll and vasculature fractions which were subsequently extracted with MTBE and analyzed for their terpene content by GC–MS. The ShTPS12-derived sesquiterpenes β-caryophyllene and α-humulene were found in the epidermis and mesophyll fractions of tomato leaves expressing the pC5-FTG construct (Table 1), while they were absent in the vasculature. Likewise, three of the ShSBS-derived sesquiterpenes, (−)-*endo*-α-bergamotene, (+)-α-santalene, and (+)-*endo*-β-bergamotene, were found in the epidermis fraction of leaves expressing the pC5-zFSG construct (Table 1), while only (+)-α-santalene and (+)-*endo*-β-bergamotene were detected in the mesophyll fraction and no ShSBS-derived sesquiterpenes were present in the vasculature of these leaves. In addition to the epidermis, mesophyll, and vasculature fractions, we also analyzed the terpene content of glandular trichomes collected from tomato leaves expressing the pC5-FTG and pC5-zFSG constructs, however, did not observe any accumulation of ShTPS12- and ShSBS-derived sesquiterpenes, respectively (Table 1). In summary, these analyses revealed that the vast majority of the ShTPS12- and ShSBS-derived sesquiterpenes, 92.99 and 92.24%, respectively, accumulate in the epidermis (Table 1) of the transiently transformed tomato leaves, thus indicating that expression of the pC5-FTG and pC5-zFSG constructs under the control of the *AtCER5* promoter indeed results in the epidermis-specific production of the engineered sesquiterpenes.

**Table 1:** Accumulation of sesquiterpenes in different tissues of tomato leaves transiently transformed with the pC5-FTG and pC5-zFSG expression constructs.

Terpenes	pC5-FTG				pC5-zFSG			
	Trichomes^*^	Epidermis^‡^	Vasculature^‡^	Mesophyll^‡^	Trichomes^*^	Epidermis^‡^	Vasculature^‡^	Mesophyll^‡^
β-caryophyllene	nd	7.68 (±1.46)	nd	0.81 (±0.22)	nd	nd	nd	nd
α-humulene	nd	2.9 (±0.73)	nd	nd	nd	nd	nd	nd
(−)-*endo*-α-bergamotene	nd	nd	nd	nd	nd	6.67 (±1.22)	nd	nd
(+)-α-santalene	nd	nd	nd	nd	nd	13.95 (±1.29)	nd	1.46 (±0.11)
(+)-*endo*-β-bergamotene	nd	nd	nd	nd	nd	10.06 (±0.87)	nd	1.12 (±0.08)

* *Terpenes were extracted from 200 glandular trichomes collected from tomato leaves*.

‡ *Epidermis, vasculature, and mesophyll fractions were prepared from the same leaves, and the amounts of terpenes (±SEM, n = 3) extracted from these fractions were normalized by the leaf surface area (pmol/cm^2^)*.

### Engineered Sesquiterpene Formation in the Epidermis Affects the Longevity and Fecundity of Aphids

**Figure 5:**
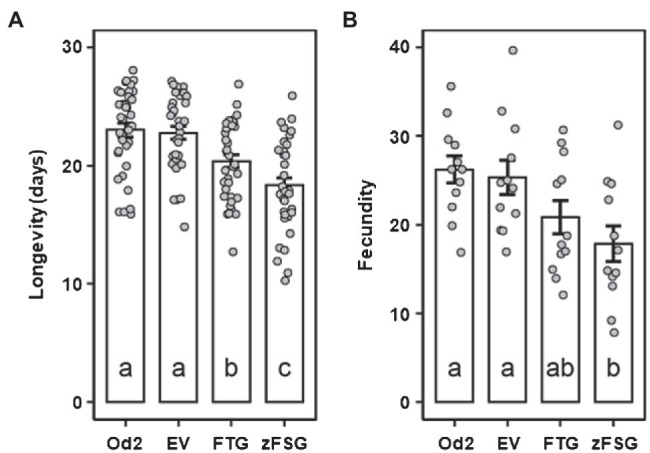
Longevity and fecundity of potato aphids on tomato leaves expressing the multicistronic constructs. Longevity **(A)** and fecundity **(B)** of *Macrosiphum euphorbiae* on leaves of the *odorless-2* tomato mutant (Od2), and leaves infiltrated with *Agrobacterium* carrying the empty pMCS vector (EV), the pC5-FTG construct, or the pC5-zFSG construct. Newly emerged aphid nymphs were arrested onto tomato leaves 2 days after *Agrobacterium* infiltration. Values for longevity (*n* = 36) and fecundity (*n* = 12) are presented as means ± SEM. Values of different leaf samples were compared by ANOVA and Tukey’s HSD test, and different letters indicate significant differences (*p* < 0.05).

As a first approach to characterize the potential of the sesquiterpene formation engineered into the leaf epidermis to affect the potato aphid (*M. euphorbiae*), we performed non-choice assays utilizing tomato leaves that transiently express the pC5-FTG and pC5-zFSG constructs. Newly emerged *M. euphorbiae* nymphs were reared in clip cages on the surface of tomato leaves that previously have been infiltrated with *Agrobacterium* carrying the pC5-FTG and pC5-zFSG constructs or the empty pMCS vector control, and their longevity and fecundity (represented by the number of offspring) were determined. Compared to the non-infiltrated *odorless-2* control, infiltration of leaves with *Agrobacterium* carrying the empty pMCS vector did not significantly affect longevity (*t* = 0.201, *p* = 0.997) or fecundity (*t* = 0.368, *p* = 0.983) of *M. euphorbiae*. In contrast, the longevity of *M. euphorbiae* on tomato leaves expressing the pC5-FTG construct (20.39 ± 0.55 days), characterized by β-caryophyllene and α-humulene production in their epidermis (Figure 4; Table 1), was significantly decreased (*t* = 5.420, *p* = 0.001) compared to that on leaves infiltrated with *Agrobacterium* carrying the empty pMCS vector (22.78 ± 0.53 days; Figure 5A). The longevity of *M. euphorbiae* (Figure 5A) on tomato leaves expressing the pC5-zFSG construct (18.33 ± 0.65 days), which accumulated the ShSBS-derived sesquiterpenes in their epidermis (Figure 4; Table 1), was also significantly decreased (*t* = 2.678, *p* = 0.048) compared to that on leaves infiltrated with the empty pMCS vector control, and even further decreased compared to that on leaves expressing the pC5-FTG construct (*t* = 2.742, *p* = 0.035). Similar effects as observed for the longevity were also found for the fecundity of *M. euphorbiae* (Figure 5B) on tomato leaves expressing the pC5-FTG and pC5-zFSG constructs, while their fecundity on leaves infiltrated with *Agrobacterium* carrying the empty pMCS vector was not significantly affected (*t* = 0.368, *p* = 0.983). The number of *M. euphorbiae* offspring was significantly reduced (*t* = 3.160, *p* = 0.015) on leaves expressing the pC5-zFSG construct (17.83 ± 2.01 nymphs) compared to leaves infiltrated with *Agrobacterium* carrying the empty pMCS vector (25.33 ± 1.94 nymphs; Figure 5B). A similar trend toward a reduced number of offspring (20.83 ± 1.86 nymphs) was observed with *M. euphorbiae* on leaves expressing the pC5-FTG construct (Figure 5B), although their fecundity was not significantly different to that of aphids on leaves infiltrated with *Agrobacterium* carrying the empty pMCS vector (*t* = 1.679, *p* = 0.347) or the pC5-zFSG construct (*t* = 1.481, *p* = 0.457).

## Discussion

It is well known that wild tomato species, such as *S. habrochaites*, have glandular trichome-derived resistance traits against numerous pests (Simmons and Gurr, 2005). In particular, some terpenes produced in the glandular trichomes of wild tomato accessions have been shown to act repellent and/or toxic against pests (Carter et al., 1989a,b; Frelichowski and Juvik, 2001; Bleeker et al., 2009, 2011a). In our previous study (Wang et al., 2020), we have identified two groups of *S. habrochaites* accessions producing β-caryophyllene/α-humulene and (−)-*endo*-α-bergamotene/(+)-α-santalene/(+)-*endo*-β-bergamotene, respectively, that significantly reduced the longevity and fecundity of *M. euphorbiae*, and also had repellent activity against the aphids. Thus, introducing these defensive sesquiterpene traits identified in *S. habrochaites* into cultivated tomato represents a logical step toward developing a novel aphid control strategy. One avenue toward achieving this goal is the classical genetic approach by crossing *S. lycopersicum* and respective *S. habrochaites* accessions, followed by backcrosses into the cultivated tomato background to obtain an introgression line carrying the *S. habrochaites* sesquiterpene trait. A near isogenic tomato line with a small *S. habrochaites* introgression carrying the *ShzFPPS* and *ShSBS* genes was previously isolated (van der Hoeven et al., 2000) and found to produce (−)-*endo*-α-bergamotene, (+)-α-santalene, and (+)-*endo*-β-bergamotene (Sallaud et al., 2009). While our assays demonstrated that introgression of the (−)-*endo*-α-bergamotene/(+)-α-santalene/(+)-*endo*-β-bergamotene formation into the cultivated tomato background indeed affected the performance and choice behavior of *M. euphorbiae*, it became obvious that the sesquiterpene levels and the resulting effects on *M. euphorbiae* were significantly lower in the introgression line (Wang et al., 2020). In contrast to the limitations of the genetic approach, metabolic engineering has been shown to offer an efficient approach to introduce the biosynthesis of terpene compounds of interest into plants (Lange and Ahkami, 2013; Vickers et al., 2014) that in addition can be steered toward specific tissues through the choice of respective promoters. To engineer high levels of terpene formation in many cases multiple biosynthetic genes have to be introduced into the host plant including MVA/MEP pathway, prenyl transferase, and terpene synthase genes. However, the introduction of multiple individual transgenes and their combination in one plant line through subsequent crosses is a time-consuming process. In addition, the stacking of several transgenes that are all expressed under the identical type of promoter bears the risk of gene silencing. In contrast, the utilization of the viral self-processing 2A sequences circumvents these problems and allows the co-expression of multiple genes under the control of a single promoter (de Felipe et al., 2006).

Here, we designed two multicistronic expression constructs, each composed of the coding sequences for a prenyl transferase, a respective *S. habrochaites* terpene synthase, and enhanced green fluorescent protein linked by short nucleotide sequences encoding the foot-and-mouth disease virus 2A self-processing oligopeptide (Figure 1). Both constructs are under the control of the *AtCER5* promoter that directs epidermis-specific gene expression (Pighin et al., 2004). Infiltration of tomato leaves with *Agrobacterium* carrying the pC5-FTG and pC5-zFSG constructs resulted in the transient expression of all three genes included in each expression construct. The RT-PCR analyses (Figure 2) demonstrated the expression of the first coding region, *AtFPPS* and *ShzFPPS*, respectively, and the third coding region, *eGFP*, from each of the two multicistronic constructs. Moreover, the expression of *eGFP* was further verified through fluorescence microscopy that detected GFP fluorescence in the epidermis (Figure 3). The formation of the expected sesquiterpenes, β-caryophyllene and α-humulene (Figure 4A), and (−)-*endo*-α-bergamotene, (+)-α-santalene, (−)-*exo*-α-bergamotene, (−)-*epi*-β-santalene, and (+)-*endo*-β-bergamotene (Figure 4B), upon leaf infiltration with *Agrobacterium* carrying the pC5-FTG and pC5-zFSG construct, respectively, provided further evidence for the expression of the prenyl transferases and terpene synthases included in these constructs. Our observation that the total sesquiterpene amounts produced in leaves expressing the plastid localized ShzFPPS and ShSBS were higher than in leaves expressing the cytosolic AtFPPS and ShTPS12 (Supplementary Table 1) is in line with earlier studies showing that the plastidic MEP pathway is often metabolically more active than the cytosolic MVA pathway (Ashour et al., 2010; Hemmerlin et al., 2012). A similar co-expression system based on viral 2A sequences has previously been used to engineer the formation of a precursor of artemisinin, a plant derived sesquiterpene lactone highly effective in the treatment of malaria, into tobacco leaves (van Herpen et al., 2010). Transient expression of a multicistronic construct, containing the reading frames for amorpha-4,11-diene synthase, 3-hydroxy-3-methylglutaryl-CoA reductase, and farnesyl diphosphate synthase linked by 2A sequences, in *Nicotiana benthamiana* leaves resulted in the formation of the artemisinin precursor amorpha-4,11-diene. Moreover, a viral 2A sequence system has been used for the co-expression of the carotenoid biosynthetic genes encoding phytoene synthase and carotene desaturase in rice endosperm to obtain an improved version of the β-carotene producing Golden Rice (Ha et al., 2010; Jeong et al., 2017). Another study (Møldrup et al., 2012) utilized the viral 2A sequence co-expression system to engineer the six-step benzylglucosinolate pathway from *A*. *thaliana* into *Nicotiana tabacum*, thus converting the resulting tobacco lines into a trap crop for the pest diamondback moth (*Plutella xylostella*).

In vegetative parts of plants, the formation of terpenes is often restricted to specific tissues, such as glandular trichomes on the leaf surface (Gershenzon and Dudareva, 2007; Zulak and Bohlmann, 2010). Therefore, the goal of this study was to test if the formation of terpenes with activity against aphids could be engineered into new vegetative tissues, specifically the epidermis, where terpenes are naturally not found. The fluorescence microscopy analyses (Figure 3) of tomato leaves infiltrated with *Agrobacterium* carrying the pC5-FTG and pC5-zFSG constructs detected GFP fluorescence exclusively in both epidermal layers, thus confirming the tissue specificity of the expression under the control of the *AtCER5* promoter. Moreover, the analysis of the terpene content (Table 1) in different tissue fractions of the tomato leaves transiently expressing the multicistronic constructs revealed that the vast majority of the ShTPS12- and ShSBS-derived sesquiterpenes is indeed produced in the epidermis. These results suggest that sufficient pools of IPP and DMAPP are available in the cytosol and plastids of these epidermis cells that can serve as substrates for the cytosolic AtFPPS2 and ShTPS12, and the plastid localized ShzFPPS and ShSBS, respectively. Although the minor amounts of sesquiterpenes (Table 1) found in the mesophyll fractions of leaves expressing the pC5-FTG and pC5-zFSG constructs are likely the consequence of contamination by epidermis cells, we cannot exclude that there might be a symplastic transport of some sesquiterpenes produced in the epidermis cells toward neighboring mesophyll cells. While to the best of our knowledge this is the first report on the metabolic engineering of terpene formation in the epidermis of leaves, there are examples of natural terpene formation in epidermis cells. The flowers of *Clarkia breweri* are strongly scented and one of the major volatile compounds emitted is the monoterpene *S*-linalool. *In situ* localization studies of the *S*-linalool synthase transcripts demonstrated that this terpene synthase is mainly expressed in the epidermal cell layers of the *C. breweri* flower petals (Dudareva et al., 1996). Likewise, both epidermal layers of the petals in rose (*Rosa* x *hybrida*) flowers were found to produce, accumulate, and emit a number of monoterpenes including geraniol, citronellol, and nerol (Bergougnoux et al., 2007).

Remarkably, the formation of the ShTPS12- and ShSBS-derived sesquiterpenes in the epidermis of tomato leaves expressing the pC5-FTG and pC5-zFSG constructs significantly affected the longevity and fecundity of *M. euphorbiae* (Figure 5). Recently, we observed similar effects on the longevity and fecundity of *M. euphorbiae* when the aphid performance was tested on the leaf surface of *S. habrochaites* accessions producing β-caryophyllene/α-humulene and (−)-*endo*-α-bergamotene/(+)-α-santalene/(+)-*endo*-β-bergamotene in their glandular trichomes (Wang et al., 2020). The fact that the effect on the performance of *M. euphorbiae* was less severe on the engineered leaves with the epidermis-specific sesquiterpene formation (Figure 5) compared to that of the glandular trichome-derived sesquiterpenes in *S. habrochaites* accessions (Wang et al., 2020) could be due to a difference in the amounts of sesquiterpenes produced. On the other hand, the reduced longevity and fecundity of *M. euphorbiae* observed in this study are clearly due to the sesquiterpene formation engineered into the leaf epidermis, since we have used the *odorless-2* mutant that is deficient in the formation of the glandular trichome-derived terpenes normally found in tomato leaves (Kang et al., 2010a; Wang et al., 2021). This result of our study is in line with previous studies which have revealed that the host plant selection by aphids is not only affected by glandular trichomes, but also by factors located in the epidermis including epicuticular lipids, cell wall barriers, and the presence or absence of certain metabolites that serve as gustatory cues upon probing (Alvarez et al., 2006; Schwarzkopf et al., 2013).

## Conclusion

In this study, we have taken a novel approach toward developing a sustainable aphid control strategy that specifically considers the feeding behavior of these piercing-sucking pests. By utilizing the viral 2A sequence system, we co-expressed two pairs of prenyl transferases and *S. habrochaites* terpene synthases under the control of the epidermis-specific *AtCER5* promoter. This metabolic engineering approach resulted not only in the exclusive accumulation of the desired sesquiterpenes in the epidermis of tomato leaves, but also significantly affected the aphid performance. Thus, the metabolic engineering of sesquiterpenes into the leaf epidermis introduced an additional layer of defense against aphids, besides the glandular trichome-derived terpenes naturally present in cultivated and wild tomato species that in particular affect the aphid choice behavior. Future metabolic engineering approaches could now also test the effects of sesquiterpene formation in other tissues relevant to aphid feeding, such as the mesophyll and phloem, by expressing the newly designed multicistronic constructs under the control of respective tissue-specific promoters. While the outcome of our transient engineering study and the aphid bioassays highlighted the potential and efficacy of the tissue-specific metabolic engineering approach described here, further detailed characterization of respective stable transgenic tomato lines and aphids feeding on them, including electrical penetration graph analysis, will be required to verify which specific stages of the aphid feeding behavior are affected by the sesquiterpene formation in the epidermis and other tissues.

## Data Availability Statement

The original contributions presented in the study are included in the article/Supplementary Material, further inquiries can be directed to the corresponding author.

## Author Contributions

MG and Y-LP conceived and designed the research. FW performed the experiments. FW, Y-LP, and MG analyzed the data. FW and MG wrote the manuscript. All authors have read and approved the final manuscript.

## Funding

This project was supported by the Agricultural and Food Research Initiative competitive grant number 2018-67014-28092 from the USDA National Institute of Food and Agriculture to MG and Y-LP. This study was also partially supported by the West Virginia Agricultural and Forestry Experiment Station Hatch Projects WVA00730 to MG and WVA00024 to Y-LP. In addition, the work of MG was supported by the Ray Marsh and Arthur Pingree Dye Professorship.

## Conflict of Interest

The authors declare that the research was conducted in the absence of any commercial or financial relationships that could be construed as a potential conflict of interest.

## Publisher’s Note

All claims expressed in this article are solely those of the authors and do not necessarily represent those of their affiliated organizations, or those of the publisher, the editors and the reviewers. Any product that may be evaluated in this article, or claim that may be made by its manufacturer, is not guaranteed or endorsed by the publisher.

## Footnotes

^1^ https://imagej.nih.gov/ij/

^2^ https://chemdata.nist.gov/mass-spc/ms-search/

## Supplementary Material

Supplementary Material 1 Mass spectra of ShTPS12-derived sesquiterpenes. Mass spectra of β-caryophyllene **(A)** and α-humulene **(B)** corresponding to peaks 1 and 2 (Figure 4), respectively, are shown. Terpenes were extracted from tomato leaves infiltrated with Agrobacterium carrying the pC5-FTG construct and were analyzed by GC–MS. Mass spectra were scanned at a range of 30–500 (m/z) after electron ionization at 70 eV.

Supplementary Material 2 Mass spectra of ShSBS-derived sesquiterpenes. Mass spectra of (−)-*endo*-α-bergamotene **(A)**, (+)-α-santalene **(B)**, (−)-*exo*-α-bergamotene **(C)**, (−)-*epi*-β-santalene **(D)**, and (+)-*endo*-β-bergamotene **(E)** corresponding to peaks 3–7 (Figure 4), respectively, are shown. Terpenes were extracted from tomato leaves infiltrated with Agrobacterium carrying the pC5-zFSG construct and were analyzed by GC–MS. Mass spectra were scanned at a range of 30–500 (m/z) after electron ionization at 70 eV.
