# Supplementary material for: Epidermis-Specific Metabolic Engineering of Sesquiterpene Formation in Tomato Affects the Performance of Potato Aphid Macrosiphum euphorbiae
Source: Front Plant Sci. 2021 Dec 22;12:793313. doi: 10.3389/fpls.2021.793313 (PMC8727598; doi:10.3389/fpls.2021.793313)
Supplement: Supplementary file 1 [file Table_1.docx]

|  |  | **pC5-FTG** | | | | | |  | **pC5-zFSG** | | | | | | | | | | |
| --- | --- | --- | --- | --- | --- | --- | --- | --- | --- | --- | --- | --- | --- | --- | --- | --- | --- | --- | --- |
| Peak | Compound * | 0 d | 3 d | 6 d | 9 d | 12 d | 15 d |  | 0 d | 3 d | | 6 d | | 9 d | | 12 d | | | 15 d |
| 1 | β-caryophyllene | nd | nd^a^ | 13.50^ab^ (±4.46) | 14.29^ab^ (±3.57) | 18.45^b^ (±3.05) | 20.93^b^ (±2.27) |  | nd | nd | | nd | | nd | | nd | | | nd |
| 2 | α-humulene | nd | nd^a^ | 2.94^ab^ (±0.94) | 4.53^ab^ (±1.76) | 7.46^b^ (±1.77) | 6.71^b^ (±0.98) |  | nd | nd | | nd | | nd | | nd | | | nd |
| 3 | (-)-*endo*-α-bergamotene | nd | nd | nd | nd | nd | nd |  | nd | nd^a^ | | 3.68^ab^ (±1.11) | | 9.11^abc^ (±2.05) | | 15.66^c^ (±3.81) | | | 13.94^bc^ (±2.33) |
| 4 | (+)-α-santalene | nd | nd | nd | nd | nd | nd |  | nd | nd^a^ | | 9.29^ab^ (±2.51) | | 23.71^ab^ (±3.88) | | 35.17^b^ (±10.32) | | | 33.45^b^ (±6.35) |
| 5 | (-)-*exo*-α-bergamotene | nd | nd | nd | nd | nd | nd |  | nd | | nd^a^ | | 1.18^ab^  (±0.3) | | 3.03^bc^ (±0.53) | | 4.60^c^ (±0.91) | 5.20^c^ (±0.53) | |
| 6 | (-)-*epi*-β-santalene | nd | nd | nd | nd | nd | nd |  | nd | | nd^a^ | | 0.41^ab^ (±0.09) | | 1.50^bc^ (±0.30) | | 3.49^d^ (±0.37) | 2.52^cd^ (±0.22) | |
| 7 | (+)-*endo*-β-bergamotene | nd | nd | nd | nd | nd | nd |  | nd | | nd^a^ | | 6.82^ab^ (±1.77) | | 19.75^abc^ (±4.53) | | 29.49^bc^ (±8.65) | 30.40^c^ (±4.83) | |

**Supplementary Table 1** Sesquiterpene production in *odorless-2* leaves after *Agrobacterium* infiltration. Leaves of the *odorless-2* tomato mutant transiently transformed with the pC5-FTG and pC5-zFSG constructs were extracted a various timepoints (0-15 days) after the *Agrobacterium* infiltration and analyzed by GC-MS. Absolute amounts of individual sesquiterpene compounds are given in pmol/cm^2^ leaf area (±SEM), n = 3. The sesquiterpene amounts at different timepoints were compared by ANOVA followed by Tukey’s HSD test and different letters in a row indicate significant differences (*P* < 0.05). nd, compound not detected. *, sesquiterpenes were identified based on their mass spectra (see Suppl. Figs. 1 & 2).
