# Supplementary figures and images for: Epidermis-Specific Metabolic Engineering of Sesquiterpene Formation in Tomato Affects the Performance of Potato Aphid Macrosiphum euphorbiae
Source: Front Plant Sci. 2021 Dec 22;12:793313. doi: 10.3389/fpls.2021.793313 (PMC8727598; doi:10.3389/fpls.2021.793313)

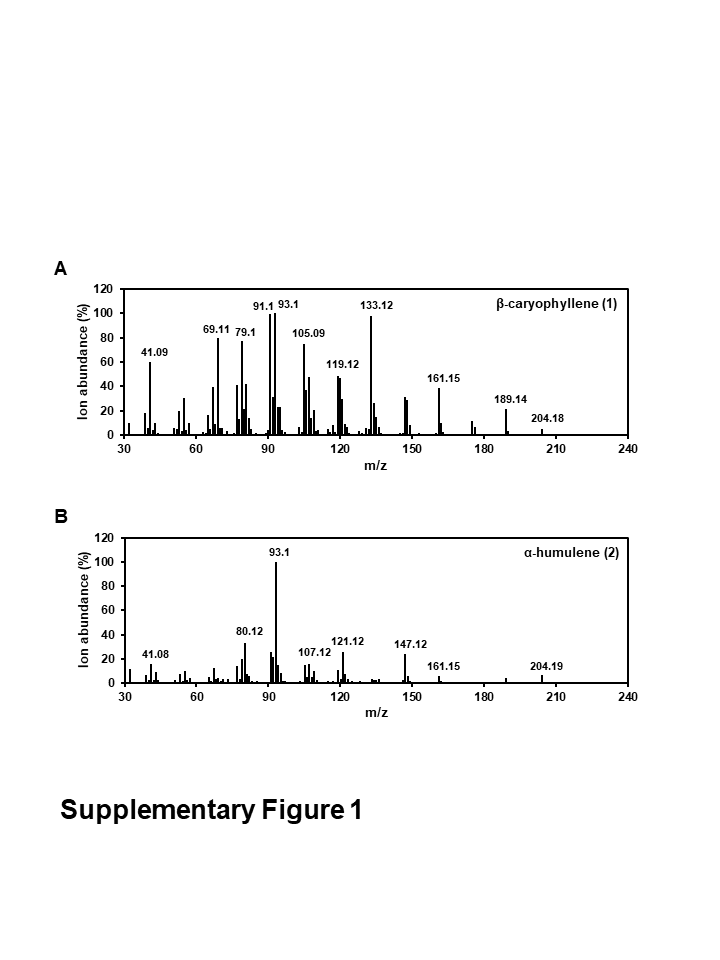

Supplement: Supplementary Material 1 — Mass spectra of ShTPS12-derived sesquiterpenes. Mass spectra of β-caryophyllene (A) and α-humulene (B) corresponding to peaks 1 and 2 (Figure 4), respectively, are shown. Terpenes were extracted from tomato leaves infiltrated with Agrobacterium carrying the pC5-FTG construct and were analyzed by GC–MS. Mass spectra were scanned at a range of 30–500 (m/z) after electron ionization at 70 eV. [file Image_1.tif]

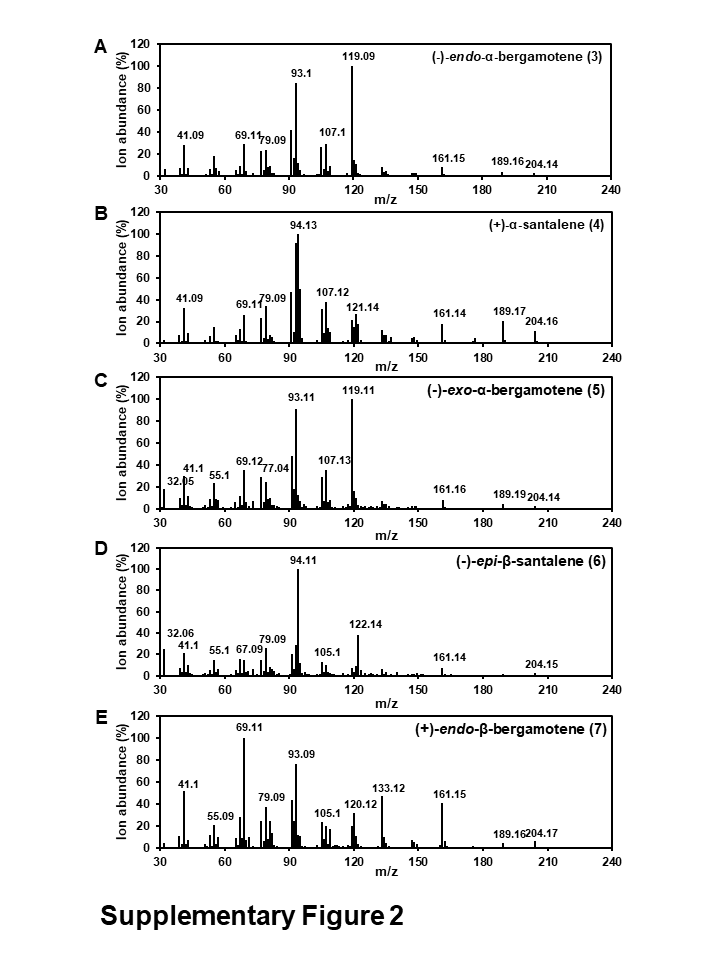

Supplement: Supplementary Material 2 — Mass spectra of ShSBS-derived sesquiterpenes. Mass spectra of (−)-endo-α-bergamotene (A), (+)-α-santalene (B), (−)-exo-α-bergamotene (C), (−)-epi-β-santalene (D), and (+)-endo-β-bergamotene (E) corresponding to peaks 3–7 (Figure 4), respectively, are shown. Terpenes were extracted from tomato leaves infiltrated with Agrobacterium carrying the pC5-zFSG construct and were analyzed by GC–MS. Mass spectra were scanned at a range of 30–500 (m/z) after electron ionization at 70 eV. [file Image_2.tif]
